# The Tropical Disease Priority Review Voucher: A Game-Changer for Tropical Disease Products

**DOI:** 10.4269/ajtmh.16-0099

**Published:** 2017-01-11

**Authors:** Jonathan Berman, Tanya Radhakrishna

**Affiliations:** 1 Fast Track Drugs and Biologics, North Potomac, Maryland.; 2 Knight Therapeutics Inc., Westmount, Canada.

## Abstract

The Neglected Tropical Disease Voucher Program is a Congressionally-mandated program intended to promote approval of products for tropical diseases because it provides spectacular financial compensation consequent to FDA approval of a priority product. Three drug approvals–artemether/lumifantrine for malaria, bedaquiline for multidrug resistant tuberculosis, miltefosine for leishmaniasis–have received Tropical Disease Vouchers to date. We give our view of the type of products that might qualify for a Tropical Disease Voucher, financial considerations in venturing capital to support product development, clinical ramifications of a successful product approval, and an overall evaluation of the Program.

## Introduction

The neglected tropical disease priority review voucher (PRV) program (“tropical disease voucher,”) is a U.S. government program intended to enlarge the number of products approved for tropical diseases in the United States.^1^ Ridley and others noted that “Infectious and parasitic diseases create enormous health burdens, but because most of the people suffering from these diseases are poor, little is invested in developing treatments.”^2^ In 2006, these academicians proposed,^2^ and in 2007, the U.S. Congress enacted a new section 524 to the Federal Food, Drug, and Cosmetic Act (21 U.S.C. 360n) that provides financial incentives for sponsors of tropical disease products. If a sponsor achieves approval of a new drug application (NDA) or biologics licensing application (BLA) for a new chemical entity (NCE) that constitutes a significant improvement for one of at least 16 listed tropical diseases, the sponsor receives a PRV which can be used for priority review of any subsequent NDA/BLA.^1^ “Priority review” means that the Food and Drug Administration (FDA) review occurs with a target of 6 months rather than the standard review period of 10 months. The PRV is transferable and can be sold for use with any other product. An approximately 4-month shorter FDA review time for a future NDA/BLA has clear monetary value providing sufficient financial incentives to develop novel tropical disease products.^3^ Note that the creation of this financial incentive costs the U.S. taxpayer essentially nothing: use of a tropical disease voucher puts drug “X” supported by the tropical disease voucher at the front of the FDA review line, with the extra voucher user fee paid by the sponsor of drug X compensating the FDA for the extra review effort.

**Figure 1.**
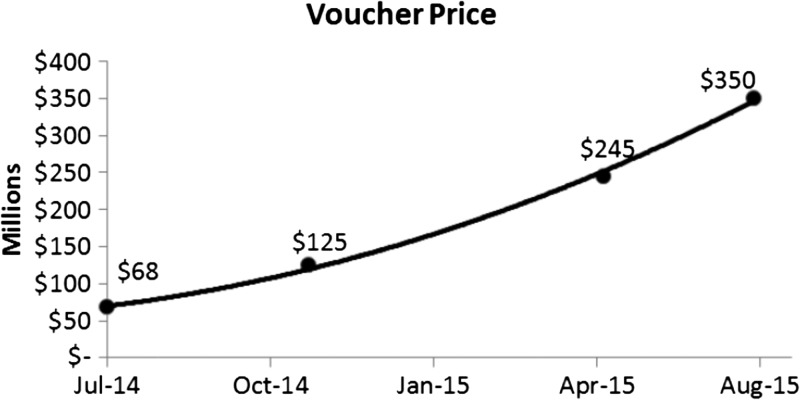
Price for which tropical disease and rare pediatric disease vouchers have sold in 2014–2015.

^4^, with the most recent price^4^ being remarkably similar to the $321 million value predicted by Ridley and others in 2006.

Between 2008 and the present (May 2016), three tropical disease vouchers have been awarded: artemether–lumefantrine for treatment of malaria (2008), bedaquiline for treatment of multidrug-resistant tuberculosis (MDR-TB; 2012), and miltefosine for treatment of leishmaniasis (2014). In addition, six vouchers for a closely related program to promote approval of drugs for rare pediatric diseases have been awarded. Five of the nine vouchers (In June 2016, Vaxchora, a vaccine against *Vibrio cholerae* serogroup O1, was approved and received a Voucher [http://www.fda.gov/NewsEvents/Newsroom/PressAnnouncements/ucm506305.htm], thus there are now a total of 10 topical disease plus rare pediatric disease vouchers.) are known to have been sold. The selling price for the four vouchers for which the data are public is shown in Figure 1

The spectacular selling price for a voucher for a tropical disease/pediatric rare disease has drawn attention to the tropical disease voucher program. We wish to comment on 1) the type of products that might qualify for a tropical disease voucher, 2) financial considerations in venturing capital to support development of a product that might qualify for a tropical disease voucher, 3) the likely clinical ramifications of a successful tropical disease NDA/BLA, and 4) an overall view of the pros and cons of the program.

## Clinical Products That May Qualify for a Neglected Tropical Disease Voucher

The tropical disease product must be an NCE that itself qualifies for priority review.^1^ These criteria have at least three important ramifications.

### Drug substance.

The NCE criterion reasonably excludes a new ether of an already-approved drug from obtaining a voucher—after artemether–lumefantrine, another dihydroartemisinin ether would not receive a voucher—but perhaps less reasonably excludes a new indication for a previously approved drug. For example, since paromomycin oral has been approved for amebiasis, neither paromomycin parenteral nor paromomycin topical would qualify for a tropical disease voucher for leishmaniasis.

### Strength of clinical dossier.

The priority review criterion means that at the time the NDA/BLA application is submitted, the FDA concludes that the product is likely to be 1) safe and effective in the treatment, prevention, or diagnosis of a disease where no marketed product exists, or 2) a significant improvement compared with marketed products.^5^ Significant improvement is further defined as increased effectiveness, substantial reduction of a treatment-limiting drug reaction, enhancement of patient compliance, or safety and effectiveness in a new subpopulation.^5^ Criterion 1, in which an NCE is compared with no treatment, is much easier to satisfy than criterion 2, which may account for the relative ease with which priority review was granted to miltefosine for leishmaniasis, for which there was no approved treatment, compared with artemether–lumefantrine for malaria which was finally granted priority review after three revisions on the basis of efficacy for a new subpopulation.

### Operational implications.

Since “priority review” means that the NDA/BLA gets reviewed on a priority basis, priority review for a tropical disease product cannot be decided until the NDA/BLA is submitted. Sponsors of a tropical disease product have an understandable desire to evaluate their chances for a tropical disease voucher a long time before filing the NDA/BLA. However, the criteria for the Fast Track, Accelerated, or Breakthrough designations^6^ during the period in which sponsor interacts with FDA before NDA/BLA filing are similar to that for priority review. As a consequence, sponsors will usually request designation for one of these expedited programs,^6^ since granting of such a designation gives confidence that the product will receive priority review when the NDA/BLA is eventually submitted.

## Financial Considerations

Although selling prices for tropical disease and rare pediatric disease vouchers have risen, investment in potentially voucher-worthy products remains limited by several risk factors.

1.  Unlike rare pediatric diseases, where low worldwide incidence may lead to unique pathways to approval, tropical diseases are generally common in some geographies and require large-scale trials. If further clinical experimentation is needed, future data may not demonstrate that the product is safe and effective and qualify for NDA/BLA approval. With this clinical risk in mind, investment is unlikely for products for which phase 3 data are not yet available.
2.  The product will qualify for an NDA/BLA, but may not be markedly superior to presently approved products. As discussed above, a product must qualify for priority review itself to earn a tropical disease voucher. If products are already approved for a disease, or if no product is approved but multiple products are in competitive development, it is difficult to predict whether the product under consideration will be viewed by FDA as a priority for that disease. The timing of each product's path to FDA approval must be understood, and the likelihood of being first to approval must be assessed.
3.  There is legislative risk around the program's very existence or the rules around its application. Calls for changes to the tropical disease voucher program may eliminate certain products from eligibility.
4.  Finally, investors will attempt to estimate the value of a future voucher sale. Despite rising prices, an investor today would not consider a $350 million selling price likely. There are currently six unused tropical disease and rare pediatric disease vouchers in the marketplace, and while some voucher holders have stated their vouchers are not for sale, the laws of supply and demand dictate that prices will fall as supply increases.^7^ Timing can affect demand. The value of a voucher is greatest for a blockbuster product which does not itself qualify for priority review, and this value is augmented if using the voucher to get to market 4 months earlier allows a company to leapfrog a competitor with a similar product. This was the case when Sanofi-Regeneron used a voucher for their PCSK9 inhibitor for familial hypercholesterolemia to beat a competitive Amgen drug to market. This perfect storm scenario does not occur with any regularity so the timing of a voucher sale is more art than science.

A further financial point is that as investors become more familiar with the program, it is unlikely that any single entity will receive the full proceeds of a voucher sale. Early-stage investors may build a share of the voucher into the return on their investment. Later-stage investors will request a further share. The product developers themselves will attempt to retain a share for their efforts. In this way, the value of a voucher is likely to be shared among several stakeholders.

## Clinical Ramifications of a Successful NDA/BLA for a Tropical Disease Product

The tropical disease voucher program permits a voucher for any NCE not marketed in the United States, whether or not it has been marketed elsewhere. Since product development costs are generally less for an NCE already marketed elsewhere compared with an NCE for which full clinical costs have yet to be expended, the tropical disease voucher program has been criticized for providing financial return unlinked to clinical product development costs.^7^,^8^ On the other hand, U.S. NDA approval of an elsewhere-marketed product may bring a more in-depth analysis of the data package than had yet been achieved, which provides extra confidence for U.S. and non-U.S. consumers alike. In addition, U.S. NDA approvals now routinely include postmarketing requirements (PMRs), which produce a wealth of clinical and clinically related data that sponsors would not otherwise generate.

For artemether–lumefantrine, which was already marketed elsewhere and for which NDA approval was based on already-completed studies, there were 14 PMRs including a study in nonimmune travelers to include overweight patients, surveillance of resistance to artemether–lumefantrine, a neurotoxicity study in juvenile rats, a clinical study on auditory function, and four clinical drug–drug interaction studies.^9^

For bedaquiline, which had not previously been marketed and for which pivotal clinical trials were apparently conducted only after the tropical disease voucher program was announced, the seven PMRs included monitoring for severe adverse effects, for resistance of MDR-TB isolates to the drug, and for drug–drug interactions with efavirenz.^10^

For miltefosine, which was marketed elsewhere and for which NDA approval was based on already-completed studies, there were six PMRs, including a clinical study of spermatogenesis and an efficacy study on heavier patients.^11^

Given the importance of immunity to cure of malaria, the artemether–lumefantrine requirement to study nonimmune patients is important for U.S. patients who are nonimmune compared with the populations that were the basis of the dossier. Oral treatment of adults generally involves a fixed dose, with heavier adults receiving less drug on an mg/kg basis than lighter adults. Given the possibility that mg/kg dose correlates with cure for both malaria and leishmaniasis, the requirement for both artemether–lumefantrine and miltefosine to evaluate efficacy in heavier patients is important for U.S. patients who are often heavier than the non-U.S. patients on whom the efficacy data were based. The requirement to further investigate key tolerance issues (presence or absence of neurotoxicity for artemether–lumefantrine and of impaired spermatogenesis for miltefosine) is of value for patients worldwide as well as in the United States.

## Overall Evaluation of the Tropical Disease Voucher Program

In favor of the program is that, at no cost to the U.S. taxpayer and without impact on FDA review of other products, tropical disease products are available for U.S. use, and increased confidence is generated for the product worldwide from an intense FDA clinical and manufacturing review and from PMR-generated data. Against the program is that two of the three tropical disease vouchers have been awarded for products that are not novel because they were already sold worldwide, and the return on investment which may either be viewed as acceptable or excessive. From the two examples of which we are aware, Retrophin received $245 million versus an investment of approximately $79 million for the voucher for cholic acid (Cholbam^®^) for rare bile acid synthesis disorders including in the pediatric population; Paladin/Knight received $125 million versus an investment of approximately $12 million for the tropical disease voucher for miltefosine for leishmaniasis. Ridley has recently reemphasized the concerns that “vouchers have been awarded for drugs that would have been developed without the incentive” and that “while the voucher program encourages innovation, it does not ensure access to drugs developed as a result of the program. Drug developers should be required to submit to FDA their plans for how to make their drugs accessible”.^7^ It may be difficult to decide whether the advantages of the PRV program outweigh the disadvantages or vice versa. Nevertheless, in 2014–2015, Congress added Ebola/filoviruses^12^ and the FDA added Chagas disease and neurocysticercosis^4^ to the list of diseases for which a tropical disease voucher may be obtained. With these actions, the U.S. government, at least, is viewing the neglected tropical disease PRV program positively.
